# Antigen-induced chimeric antigen receptor multimerization amplifies on-tumor cytotoxicity

**DOI:** 10.1038/s41392-023-01686-z

**Published:** 2023-12-08

**Authors:** Yan Sun, Xiu-Na Yang, Shuang-Shuang Yang, Yi-Zhu Lyu, Bing Zhang, Kai-Wen Liu, Na Li, Jia-Chen Cui, Guang-Xiang Huang, Cheng-Lin Liu, Jie Xu, Jian-Qing Mi, Zhu Chen, Xiao-Hu Fan, Sai-Juan Chen, Shuo Chen

**Affiliations:** 1 Shanghai Institute of Hematology, National Research Center for Translational Medicine, State Key Laboratory of Medical Genomics, Ruijin Hospital Affiliated to Shanghai Jiao Tong University School of Medicine, Shanghai, 200025 China; 2 Shanghai Institute for Advanced Immunochemical Studies and School of Life Science and Technology, ShanghaiTech University, Shanghai, 201210 China; 3 Department of Hematology, Second Hospital of Dalian Medical University, Dalian, 116023 China; 4 National Facility for Protein Science Shanghai, Shanghai Advanced Research Institute, Chinese Academy of Sciences, Shanghai, 201210 China; 5 Legend Biotech China, Nanjing, 211112 China; 6 Shanghai Immune Therapy Institute, Shanghai Cancer Institute, State Key Laboratory of Oncogenes and Related Genes, Renji Hospital Affiliated to Shanghai Jiao Tong University School of Medicine, Shanghai, 200127 China; 7 Present Address: Wondercel Biotechnology, Shenzhen, 518052 China

**Keywords:** Haematological cancer, Immunotherapy

## Abstract

Ligand-induced receptor dimerization or oligomerization is a widespread mechanism for ensuring communication specificity, safeguarding receptor activation, and facilitating amplification of signal transduction across the cellular membrane. However, cell-surface antigen-induced multimerization (dubbed AIM herein) has not yet been consciously leveraged in chimeric antigen receptor (CAR) engineering for enriching T cell-based therapies. We co-developed ciltacabtagene autoleucel (cilta-cel), whose CAR incorporates two B-cell maturation antigen (BCMA)-targeted nanobodies in tandem, for treating multiple myeloma. Here we elucidated a structural and functional model in which BCMA-induced cilta-cel CAR multimerization amplifies myeloma-targeted T cell-mediated cytotoxicity. Crystallographic analysis of BCMA–nanobody complexes revealed atomic details of antigen–antibody hetero-multimerization whilst analytical ultracentrifugation and small-angle X-ray scattering characterized interdependent BCMA apposition and CAR juxtaposition in solution. BCMA-induced nanobody CAR multimerization enhanced cytotoxicity, alongside elevated immune synapse formation and cytotoxicity-mediating cytokine release, towards myeloma-derived cells. Our results provide a framework for contemplating the AIM approach in designing next-generation CARs.

## Introduction

Multimerization, such as dimerization and oligomerization, of cell surface receptors induced by interaction with cognate extracellular signalling molecules is fundamental to many cell–cell communication pathways.^1^ Examples include receptor tyrosine kinases that are activated by ligand-induced dimerization,^2^ and members of the tumor necrosis factor (TNF) receptor superfamily which are triggered by trimerization.^3^ The octameric T cell receptor (TCR) complex represents a more intricate exemplar in which antigen engagement ultimately leads to the phosphorylation of activation motifs in the CD3ζ subunit dimer.^4^ This octameric TCR complex has been streamlined into a single-pass transmembrane protein termed chimeric antigen receptor (CAR) that is the cornerstone of many engineered T cell-based immunotherapies.^5^ A CAR integrates an extracellular antibody region to recognize its target cell surface antigen and intracellular CD3ζ activation motifs. Efforts focusing on intracellular costimulatory modules spearheaded several generations of CAR evolution,^5–7^ yet this approach appears to be reaching the limit of what a single polypeptide chain could offer. Inspired by ligand-induced multimerization found in nature, we hypothesized that antigen-induced multimerization mediated by the extracellular antibody domain could be leveraged in CAR design to enhance T cell cytotoxicity towards rogue cells earmarked by cognate cell surface antigens.

Notably, antigen-independent,^8,9^ antibody-crosslinked,^10–12^ or soluble ligand-mediated^13^ multimerization was associated with CAR activation and augmented signaling. Antigen-independent dimerization and clustering were observed for many single-chain variable fragments (scFv)-based CARs,^8,9^ and found to trigger constitutive tonic signaling that led to early exhaustion of engineered T cells, especially for scFvs coupled with the CD28 costimulatory domain.^8^ Substitution with the 4-1BB module auspiciously ameliorated T cell exhaustion.^8^ Intriguingly, 4-1BB costimulated tonic signaling actuated by antigen-independent CAR dimerization seemed to improve clinical efficacy.^9^ It is, therefore, conceivable, based on these previous observations, that antigen-induced multimerization for CAR design could potentially enhance communication specificity, safeguard receptor activation, and facilitate amplification of signal transduction for engineered T cells.

**Fig. 1:**
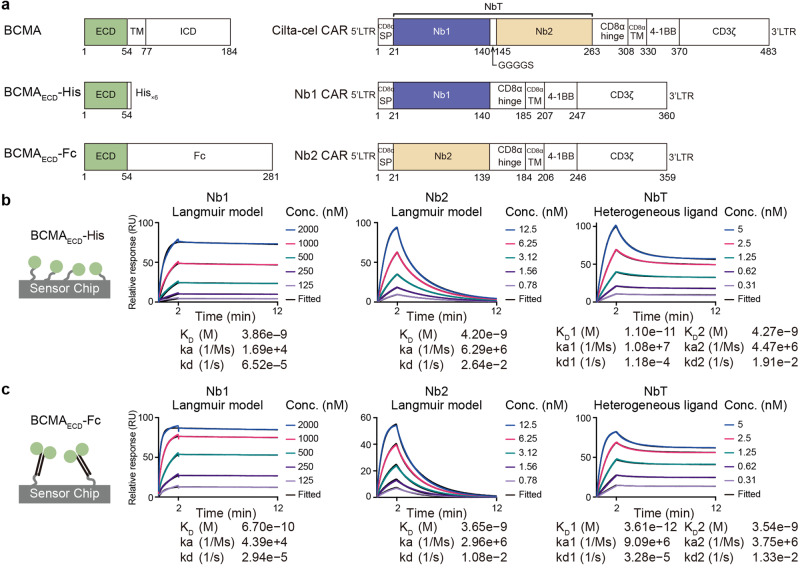
Synergistic BCMA interactions with the cilta-cel tandem of nanobodies. **a** Schematic diagrams of BCMA and CAR constructs used in this study. ECD extracellular domain, TM transmembrane region, ICD intracellular domain, LTR long terminal repeat, SP signal peptide, Nb1, Nb2, and NbT, nanobody 1, 2, and tandem. Regarding the CAR constructs, the SP, hinge, and TM domains are derived from human CD8α. **b**, **c** Characteristics of BCMA–nanobody binding assayed by surface plasmon resonance (SPR). SPR sensorgrams of nanobodies as the analytes (concentration [nM] indicated) over immobilized BCMA_ECD_-His (**b**) or BCMA_ECD_-Fc (**c**) as the ligands. Binding models (Langmuir or heterogeneous ligand) used for fitting (black overlay) are denoted above and the derived affinities and kinetics are shown below each chart

B-cell maturation antigen (BCMA) is a member of the TNF receptor superfamily^3^ (Fig. 1a left). As such, binding of TNF-like trimeric ligands—a proliferation-inducing ligand (APRIL) and B cell activating factor (BAFF)—induces trimerization and activation of BCMA, promoting B-cell proliferation and survival.^3,14,15^ BCMA is preferentially expressed on mature B lymphocytes, and significantly elevated on multiple myeloma (MM) cells alongside soluble APRIL and BAFF in MM patients.^15–18^ Hence, BCMA represents a hotspot target for therapeutic development against MM.^19,20^ MM is a common hematological malignancy, whose standard care includes proteasome inhibitors, immunomodulatory drugs, antibodies, steroids, chemotherapy, and autologous hematopoietic stem cell transplantation, but is hardly curable.^21^ Recent clinical options to treat relapsed or refractory MM (RRMM) include BCMA-directed CAR T therapies and bispecific T cell engagers.^21–26^ We co-developed ciltacabtagene autoleucel (cilta-cel), which hinges on a CAR guided by two BCMA-targeted nanobodies in tandem (referred to as nanobody tandem hereafter) to treat MM (Fig. 1a right). Nanobodies, including V_H_H (variable region of the heavy chain of heavy chain only antibody) variants used in cilta-cel, are single immunoglobulin-fold antibodies of ~120 residues each. Cilta-cel is the first nanobody-based CAR T therapy approved by the US Food and Drug Administration (FDA). Thanks to encouraging clinical results in treating RRMM,^23,24,27–30^ cilta-cel is gaining momentum in regulatory approval worldwide. In this study, we set out to delineate the structural and functional mechanism of BCMA engagement by the cilta-cel nanobody tandem in myeloma-targeted CAR activation and T cell cytotoxicity, in order to guide the design of future CARs.

## Results

### A synergistic anti-BCMA nanobody tandem

We generated a panel of nanobodies against BCMA, and selected a couple with the highest affinities from the initial screen (Nb1 and Nb2) to arrange in tandem in the extracellular domain of the CAR used in cilta-cel (Fig. 1a right). BCMA interactions with the individual nanobodies and the nanobody tandem (NbT) were further characterized using surface plasmon resonance (SPR) (Fig. 1b, c). Both Nb1 and Nb2 displayed similar, nanomolar affinities (3.9 nM for Nb1 and 4.2 nM for Nb2) to the monomeric, His-tagged BCMA extracellular domain (BCMA_ECD_-His) (Fig. 1a and Supplementary Fig. 1a) yet markedly different kinetics—Nb1 showed much slower association and dissociation than Nb2 (Fig. 1b). The SPR data for the nanobody tandem did not fit the 1:1 Langmuir binding model well, possibly due to two distinct BCMA-binding sites on the tandem (Supplementary Fig. 1b). Instead, the heterogeneous ligand model improved the fitting substantially (Fig. 1b and Supplementary Fig. 1b), and indicated a binding site with characteristics similar to Nb2 and a considerably stronger, picomolar (11 pM) BCMA interaction site (Fig. 1b). Trimerization is critical for BCMA signaling,^3^ and ligand affinities to BCMA were reported to depend on BCMA multimerization.^31^ We used dimerized Fc-tagged BCMA_ECD_ (Fig. 1a and Supplementary Fig. 1a) to approximate the biology. While Nb2 affinity to BCMA_ECD_-Fc (3.7 nM) enhanced merely slightly relative to BCMA_ECD_-His, Nb1 showed a more pronounced increase (0.67 nM), indicating a greater avidity effect, leading to stronger binding for the NbT (3.6 pM for the higher-affinity site) (Fig. 1c and Supplementary Fig. 1b). Therefore, combining the nanobodies in a tandem showed synergy in BCMA binding, resulting in an avidity-driven, picomolar antigen recognition module.

### Crystal structures of the BCMA–nanobody complexes

**Fig. 2:**
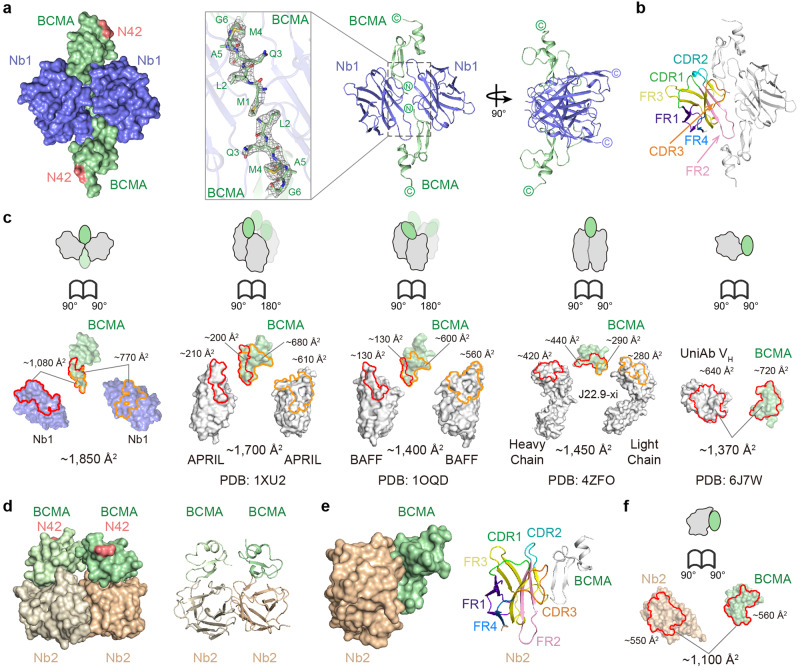
Crystallographic analysis of the BCMA–nanobody complexes. **a** Surface and cartoon representation of the crystal structure of Nb1 in complex with BCMA_ECD_. Nb1 is colored blue and BCMA green. The putative *N*-linked glycosylation sites of BCMA (N42) are highlighted in pink. Inset, electron density (2***F***_**O**_ − ***F***_**C**_ map contoured at 1 σ) of the amino (N)-terminal region of BCMA is shown as gray mesh. **b** Cartoon representation of the Nb1 complex with the complementarity-determining region (CDR) 1 of Nb1 colored in green, CDR2 in cyan, and CDR3 in orange while framework region (FR) 1 in purple blue, FR2 in pink, FR3 in yellow, and FR4 in marine. **c** Open-book views of BCMA complexes showing interfaces with antibodies and ligands. Interface residues are outlined in red or orange. **d**, **e** Surface and cartoon representation of the crystal structure of Nb2 in complex with BCMA_ECD_. Nb2 chains are colored wheat and light wheat while BCMA molecules pale green and praseodymium green for the observed two pairs in the asymmetric unit (**d**). The putative *N*-linked glycosylation sites of BCMA are highlighted. For the cartoon representation in (**e**), the Nb2 CDRs and FRs are color-coded as in (**b**). **f** An open-book view showing the interface between Nb2 and BCMA_ECD_. The interface residues are outlined in red

We next endeavored to uncover the structural basis of such synergistic, avidity-enhanced BCMA interactions with the cilta-cel tandem of nanobodies. We tried extensively to crystallize and characterize using electron microscopy (EM) the NbT–BCMA_ECD_ complex but were unable to arrive at the atomic structure, possibly due to the flexibility of the linker between the two nanobodies (Fig. 1a right) and, by extension, the resulting complex. Nonetheless, we solved the crystal structures of the individual nanobody–BCMA complexes (Fig. 2a–f and Supplementary Table 1).

The structure of the Nb1–BCMA_ECD_ complex was determined at 2.4-Å resolution, and the crystallographic asymmetric unit contained two copies of the complex arranged in a parallelogram shape, such that the membrane-proximal carboxyl (C)-termini of each component are on the opposite sides of the parallelogram plane (Fig. 2a and Supplementary Table 1). Synthesized BCMA_ECD_ without glycosylation crystalized with Nb1, yet the putative *N*-linked glycosylation is distal to Nb1 interactions and thus not likely to affect the observed structure (Fig. 2a). The two BCMA_ECD_ molecules are organized head-to-head, and the amino (N)-terminal moiety of each antigen polypeptide is embraced by two Nb1s burying a total solvent-accessible surface area of ~1,850 Å^2^ at the interfaces (Fig. 2a–c). The primary BCMA_ECD_–Nb1 interface involves the complementarity-determining regions (CDRs) 2 and 3 of Nb1 and the β hairpin of BCMA (Fig. 2b and Supplementary Fig. 2a), accounting for ~1080 Å^2^ in buried surface area (Fig. 2c). At the secondary interface, the framework region 2 (FR2) of Nb1 plays a major role and contacts the N-terminal loop of BCMA, contributing ~770 Å^2^ in buried surface area (Fig. 2a–c and Supplementary Fig. 2a). As shown in Fig. 2c, the combined Nb1 interface observed for each BCMA_ECD_ is slightly larger than the APRIL complex (Protein Data Bank [PDB] accession code: 1XU2; ~1700 Å^2^),^32^ and significantly greater than BCMA complexes with BAFF (PDB: 1OQD; ~1400 Å^2^),^33^ conventional antibodies (for instance PDB: 4ZFO; ~1450 Å^2^),^34^ and other nanobodies (for example PDB: 6J7W; ~1370 Å^2^).^35^

There is minimal contact between the two BCMA_ECD_ polypeptides (84 Å^2^), and limited interaction between the two Nb1 molecules (360 Å^2^) which seems mostly induced by BCMA_ECD_ (Fig. 2a, b and Supplementary Fig. 2b, c). Observation during purification using size-exclusion chromatography (SEC) suggested Nb1 as a monomer in solution, but data for BCMA_ECD_ were less conclusive, due to SEC limitations (Supplementary Fig. 3a, b). Likewise, the SEC results for the Nb1–BCMA_ECD_ complex hinted at a possible 2:2 stoichiometry (Supplementary Fig. 3c). The Nb1–BCMA_ECD_ structure (Fig. 2a–c) hence uncovered the assembly of BCMA-induced, Nb1-mediated dimer-of-dimers tetramerization, and the basis of the avidity-driven affinity increase measured using SPR (Fig. 1b, c).

In parallel, we solved the crystal structure of the Nb2–BCMA_ECD_ complex at the resolution of 2.7 Å (Fig. 2d–f and Supplementary Table 1). Although the asymmetric unit again contained two copies of the complex, each BCMA_ECD_ contacts one Nb2 exclusively in this structure (Fig. 2d). Herein, CDR2, CDR3, FR2, and FR3 of Nb2 interact with the β hairpin and the ensuing α-helical turn of BCMA, with a total of ~1100 Å^2^ buried at the interface (Fig. 2e, f). Again, the putative *N*-linked glycosylation is oriented away from the nanobody (Nb2), and therefore should not impact the observed structure (Fig. 2d). The BCMA_ECD_–BCMA_ECD_ interface seems small here (150 Å^2^), while the Nb2–Nb2 contact is relatively extensive (~1,370 Å^2^) but appears not significant enough to mediate nanobody dimerization (Fig. 2d). Consistent with the structural analysis, the SEC results showed monomeric Nb2 in solution (Supplementary Fig. 3b) and indicated, most likely, a 1:1 stoichiometry (Supplementary Fig. 3c).

The crystallographic analysis collectively revealed the structural potential for BCMA-induced, Nb1-mediated dimerization of the cilta-cel nanobody tandem.

### Interdependent BCMA–nanobody multimerization

**Fig. 3:**
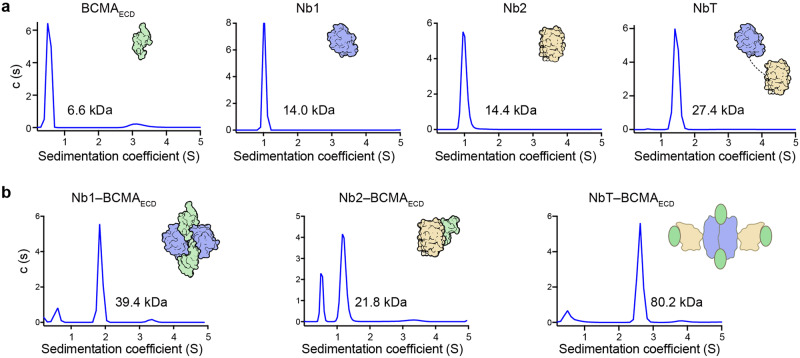
Interdependent BCMA–nanobody multimerization in solution. **a** Analysis of the molecular masses of the BCMA_ECD_, nanobodies, and NbT in solution using sedimentation velocity analytical ultracentrifugation (SV-AUC). The derived molecular weights are indicated for the corresponding peaks. Cartoon illustrations reflect our interpretation of the stoichiometries. **b** Molecular masses of the BCMA_ECD_ complexes assayed by SV-AUC. Excess BCMA_ECD_ was used to form the nanobody complexes

**Fig. 4:**
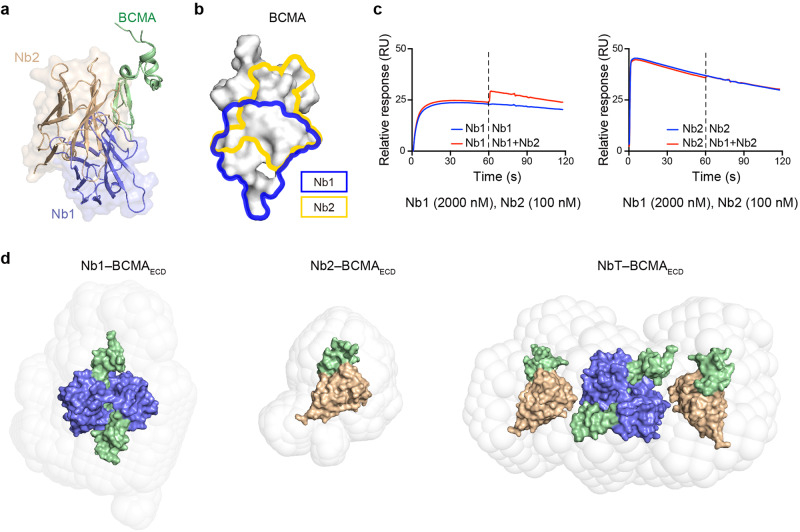
A model for the BCMA–NbT complex. **a** Superposition of the Nb1 and the Nb2 complexes based on BCMA_ECD_. The secondary interface observed in the Nb1–BCMA_ECD_ structure is omitted for clarity. **b** Nanobody epitopes mapped on the BCMA_ECD_ surface. Residues in contact with Nb1 are outlined in blue while those in contact with Nb2 in yellow. **c** Competition for BCMA binding between Nb1 and Nb2 assayed using SPR. Fc-tagged human BCMA_ECD_ was immobilized onto a Protein A sensor chip as the ligand. Nb1 (2000 nM) or Nb2 (100 nM) was loaded with BCMA_ECD_-Fc for 1 min, followed by a mixture of Nb1 (2000 nM) and Nb2 (100 nM). **d** Small-angle X-ray scattering (SAXS) envelopes and corresponding models of Nb1 (left), Nb2 (middle), and NbT (right) based on the crystal structures of the Nb1–BCMA_ECD_ and the Nb2–BCMA_ECD_ complexes

We then employed sedimentation velocity analytical ultracentrifugation (SV-AUC) to more definitively investigate the stoichiometry of the BCMA–nanobody interactions (Fig. 3a, b) owing to SEC limitations and the resulting ambiguities (Supplementary Fig. 3a–c). BCMA_ECD_ (calculated molecular weight [Mw] of 5.9 kilodalton [kDa]), Nb1 (13.9 kDa), Nb2 (13.6 kDa), and the tandem (27.0 kDa) were further analyzed to be predominantly monomeric in solution using SV-AUC (Fig. 3a). Of note, while the Nb2–BCMA_ECD_ complex was apparently 1:1 in SV-AUC (21.8 kDa), the Nb1 complex seemed to be 2:2 in stoichiometry (39.4 kDa) (Fig. 3b), which corresponded well to our SPR results using monomeric BCMA_ECD_-His and dimerized BCMA_ECD_-Fc in relation to avidity (Fig. 1b, c) and corroborated our crystallographic findings (Fig. 2a, d, and e). We measured a Mw of 80.2 kDa for the NbT–BCMA_ECD_ complex (Fig. 3b right). The superposition of our nanobody complexes based on BCMA shows considerable steric clashes (Fig. 4a). The nanobody epitopes on BCMA also overlap significantly (Fig. 4b). Furthermore, we tested the competitive binding between the nanobodies to BCMA using SPR (Fig. 4c). In this assay, the nanobodies displayed mutually exclusive binding to BCMA (Fig. 4c). Therefore, we expect each NbT to bind two BCMA_ECD_, and the most likely scenario is that 2 NbT molecules associated with 4 BCMA_ECD_ polypeptides in solution. We thus surmise that BCMA induces dimerization of NbT, through Nb1.

### Modelling the BCMA–NbT complex

To visualize the overall organization of the NbT–BCMA_ECD_ complex, given the aforementioned technical difficulties encountered with crystallography and EM, we used small-angle X-ray scattering (SAXS) to reconstruct the structural envelope, albeit at relatively low resolution (Fig. 4d). The SAXS data for the individual nanobody complexes were consistent with the crystal structures (Fig. 4d, Supplementary Fig. 4a, b, and Supplementary Table 2). The reconstructed envelope for the NbT–BCMA_ECD_ complex suggested a butterfly-shaped structure (Fig. 4d right). Due to the steric clashes and overlapping epitopes in relation to the BCMA nanobodies (Fig. 4a, b), we built a model integrating our crystal structures as three rigid bodies (the hetero-tetramer of the Nb1 complex and two copies of the Nb2 complex) in the SAXS envelope to illustrate the overall organization of the NbT–BCMA_ECD_ structure (Fig. 4d and Supplementary Fig. 4c).

### Disruption of BCMA signaling

**Fig. 5:**
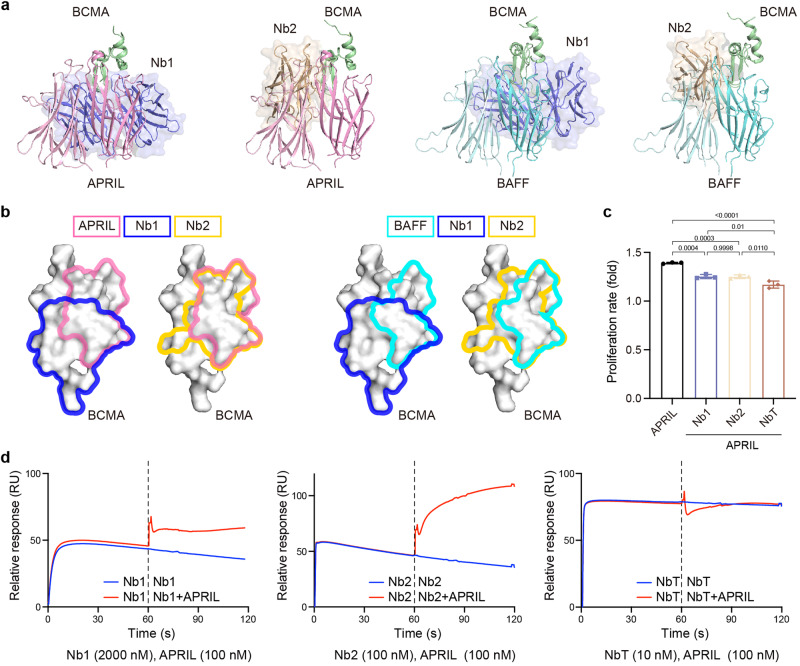
Soluble nanobodies inhibit ligand binding and signaling of BCMA. **a** Superposition of the APRIL (PDB: 1XU2) and BAFF (PDB: 1OQD) complexes with our nanobody complexes based on BCMA. Neighboring APRIL chains that sandwich BCMA are colored pink and light pink, while BAFF chains are in aquamarine and pale cyan. **b** APRIL and BAFF interface compared to Nb1 and Nb2 epitopes on BCMA. Color coding is explained above the BCMA models. **c** Nanobodies inhibit APRIL-induced MM.1S proliferation. Data are represented as means and standard errors of the mean (SEM) of *n* = 3 replicates per group, the one-way analysis of variance (ANOVA) followed by Tukey’s multiple comparisons test was used to assess the differences among different groups. Data are represented as mean ± SEM. **d** Nb1 (2000nM; left), Nb2 (100 nM; middle), and NbT (10 nM; right) were probed for competitive binding against APRIL using SPR

Superposition of our nanobody–BCMA structures with the APRIL (PDB: 1XU2) and the BAFF (PDB: 1OQD) complexes indicated substantial steric clashes as well as epitope overlap between the nanobodies and the natural ligands (Fig. 5a, b). Increased levels of secreted APRIL and BAFF in addition to MM cell-surface BCMA are common in MM patients,^16,18^ and we observed APRIL-induced proliferation of MM cell line MM.1S (Fig. 5c). The proliferation was markedly inhibited by the soluble nanobodies (*P* < 0.001 for all three purified nanobody constructs), most notably by the tandem (*P* < 0.05 against the individual nanobodies) (Fig. 5c). Under the same experimental conditions, MM.1S proliferation stimulated by BAFF was rather muted (Supplementary Fig. 5a), as it was well documented to represent a lower-affinity ligand and weaker activator of BCMA.^31^ In competitive SPR, the individual nanobodies seemed to attenuate APRIL interaction with BCMA, and the tandem apparently blocked the binding (Fig. 5d). In the case of BAFF, more complete inhibition was observed (Supplementary Fig. 5b). We reason that inhibition of ligand-induced proliferation of MM cells by the soluble nanobodies was predominantly due to competitive binding, which scaled well with affinities. Yet it is tempting to speculate that the tetramerization of the full-length BCMA transmembrane receptor by the tandem contributed to the inhibition of cell proliferation, through disruption of BCMA trimerization required for signal transduction.

### BCMA-induced CAR multimerization and cytotoxicity

**Fig. 6:**
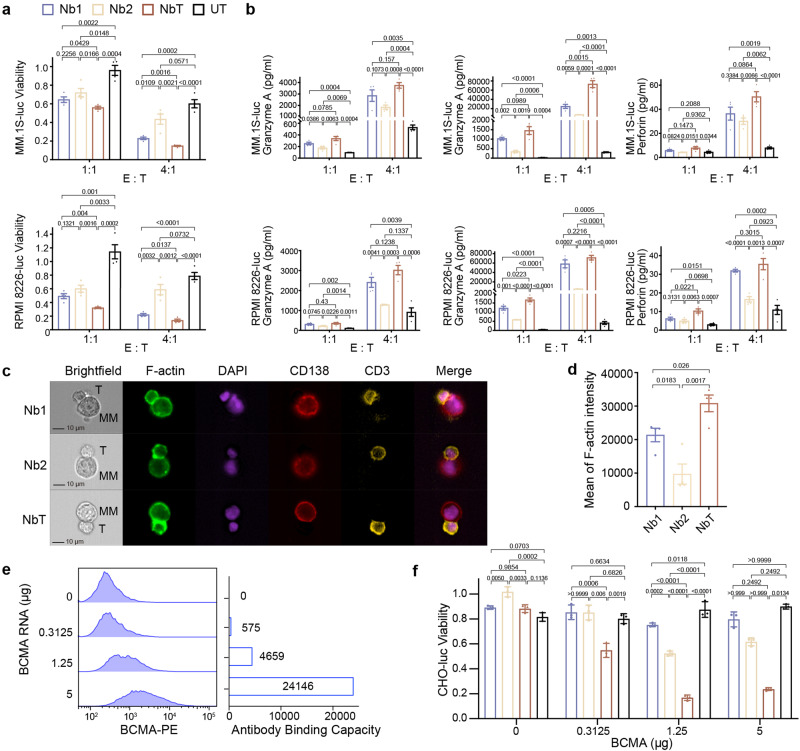
Antigen-induced CAR dimerization enhances on-tumor cytotoxicity. **a** Viability of MM.1S-luc (upper) and RPMI 8226-luc cells (lower) co-cultured with T cells transduced with the indicated nanobody CAR constructs compared to untreated (UT) control T cells (color coding at the top of the panel) for 24 h at the effector cell: target cell (E: T) ratios of 1:1 and 4:1. *n* = 4 replicates per group. Unpaired two-tailed Student’s *t*-test was performed on each grouped sample without adjustments for multiple comparisons. **b** Granzyme A, granzyme B, and perforin levels in the supernatant were measured. *n* = 4 replicates per group. Unpaired two-tailed Student’s *t*-test was performed on each grouped sample without adjustments for multiple comparisons. **c** Representative imaging flow cytometry micrographs of tested CAR T–MM.1S interactions at ×40 magnification. **d** CAR T cells were assessed for mean fluorescence intensity of F-actin at the immune synapse. *n* = 4 replicates per group. Unpaired two-tailed Student’s *t*-test was performed without adjustments for multiple comparisons. **e** Evaluation of cell-surface BCMA levels on CHO-luc cells. Escalating amounts of BCMA RNA were delivered by electroporation and the antibody binding capacity was assessed using an anti-BCMA antibody conjugated with phycoerythrin. **f** Viability of CHO-luc cells electroporated with varying BCMA amounts as measured in (**e**) while being co-cultured with the indicated CAR T cells for 24 h at the effector cell: target cell (E: T) ratios of 4:1. The one-way analysis of variance (ANOVA) followed by Tukey’s multiple comparisons test or the Kruskal-Wallis test followed by Dunn’s multiple comparisons correction was performed to assess the differences among different groups, *n* = 3 replicates per group. All data are represented as mean ± SEM

To compare CAR-mediated T cell cytotoxicity towards myeloma cells, we produced T cells engineered with otherwise identical CAR constructs incorporating either Nb1, Nb2, or the tandem along with untransduced T (UT) lymphocytes (Figs. 1a, 6a, and Supplementary Fig. 6a). CD8α forms a disulfide-linked dimer, mediated by the membrane-proximal cysteine residue in the hinge region.^36^ We tested our nanobody CAR constructs, each using a CD8α hinge, for disulfide-linked dimers (Supplementary Fig. 6b), and predict that all our CAR constructs likely express on the cell surface mainly as disulfide-bonded dimers. To assess the cytotoxicity, MM.1S-luc and RPMI 8226-luc, MM cell lines constitutively expressing the firefly luciferase were used as target cells. Nb1, Nb2, or the tandem CAR T cells were co-incubated with target cells at 1:1 and 4:1 effector: target (E: T) ratio for 24 h (Fig. 6a and Supplementary Fig. 6a). Compared to UT cells, all three CAR T cell types elicited cytotoxicity against MM cells: the tandem CAR T cells displayed the most robust killing (*P* < 0.05 against the single-nanobody constructs under all tested conditions; *P* < 0.01 at the 1:1 ratio for RPMI 8226 cells and at the 4:1 ratio for MM.1S cells and against the Nb2 construct for RPMI 8226 cells), followed closely by Nb1 CAR T cells (at the 4:1 ratio, *P* < 0.05 for MM.1S and *P* < 0.01 for RPMI 8226) (Fig. 6a and Supplementary Fig. 6a). Since Nb1 and Nb2 exhibited similar affinities to BCMA (Fig. 1b), we attributed stronger cytotoxicity to Nb1-mediated CAR multimerization revealed using biophysics (Figs. 2a, e and 3b). Production of toxicity-associated cytokines was measured from the supernatant of the co-cultures (Fig. 6b). The tandem CAR T cells generated high levels of granzyme A, granzyme B, and perforin (Fig. 6b), trailed by Nb1 CAR T cells, mirroring our toxicity data (Fig. 6a). These results suggest that BCMA-induced CAR multimerization amplifies cytotoxicity, and championed by the nanobody tandem design likely due to higher antigen affinity and possibly antigen multimerization.

### Immune synapses

CAR T immune synapse (IS) is the interface between CAR T and target cells, characterized by close apposition of two cells and direct secretory activities. The most important IS biological activity lies in the output of cytotoxic granules, including perforin and granzymes, to the target cell.^37^ Using imaging flow cytometry, IS quality assessment and quantification were performed (Fig. 6c, d). F-actin, a specific marker for cytoskeleton, was used as an indicator of the presence of IS (Fig. 6c, d). The tandem CAR T cells showed significantly higher F-actin levels at IS (*P* < 0.05 against the single-nanobody constructs), followed by Nb1 cells (*P* < 0.05 against the Nb2 construct), indicating elevated IS formation (Fig. 6c, d).

### Killing cells with low BCMA levels

MM cell lines, for example, MM.1S and RPMI 8226, display high cell-surface BCMA levels (Supplementary Fig. 6c), and are widely used as cellular models of MM. However, on-target, off-tumor toxicity is frequently observed for CAR T, and a major hurdle for application, especially in solid tumors. CAR T cytotoxicity towards normal cells with low antigen levels is a key factor. Therefore, we examined the cytotoxicity of the three CAR T cell types on target cells with a range of relatively low surface BCMA levels (Fig. 6e, f). Following the establishment of Chinese hamster ovary (CHO) strain stably-transduced with the firefly luciferase (CHO-luc), escalating amounts of BCMA transcripts were delivered into the CHO-luc cells by electroporation (Fig. 6e left). The abundance of cell-surface BCMA was quantified using a flow cytometry-based BCMA antibody binding capacity assay (Fig. 6e right). Compared to the two single nanobody CAR T populations, tandem CAR T cells showed a statistically more robust killing effect on cells electroporated with low BCMA levels (*P* < 0.01 for 0.3125 μg and *P* < 0.001 for 1.25 μg) (Fig. 6f), owing possibly to the enhanced affinity. At the spectrum of low BCMA levels tested in this assay, the Nb2 CAR T largely demonstrated greater cytotoxicity than the Nb1 counterpart that was on par with untransduced T cells (Fig. 6f). In SPR, we measured overall comparable BCMA affinities for the individual nanobodies, but Nb1 binding showed a stronger avidity effect, i.e., its affinity for BCMA increases with antigen levels (Fig. 1b, c). Apparently, the relative cytotoxicities of Nb1 and Nb2 CAR T towards cells with low BCMA levels (Fig. 6f) were considerably reversed compared to killing of MM cell lines (Fig. 6a).

## Discussion

**Fig. 7:**
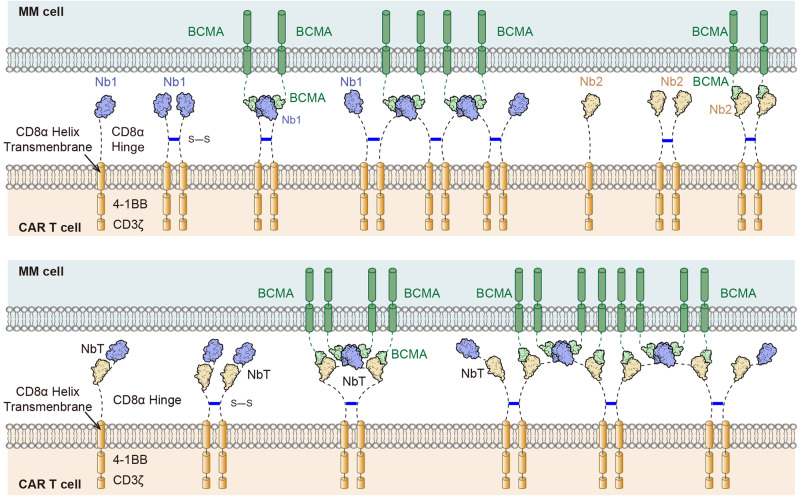
Model of BCMA-induced nanobody CAR multimerization

Our data characterize cilta-cel, to our knowledge, as the first structural example of cell surface antigen-induced CAR multimerization in T cell-based cancer immunotherapy (Fig. 7). Balancing on-tumor and off-tumor cytotoxicity by engineered T cells is a delicate act for CARs that target non-mutated, cancer-associated antigens,^5–7,38^ and antigen-induced multimerization could be an integrated modality to shift the balance. The CD19 paradigm and the more recent BCMA success for treating B-cell cancers with CAR T pivoted on high levels of these antigens on cancerous cells relative to normal tissues, which are confined to the B-cell lineage.^5,23^ On-target, off-tumor toxicity of CAR T therapies including cilta-cel towards these B cells could be mitigated with immunoglobulin replacement therapy.^5,23^ Such cell surface antigens have proven challenging to identify for solid tumors, and thus CARs would have to distinguish target solid tumor cells with high antigen density, amplifying T cell response upon engagement, from cells with lower antigen levels. Notably, multiple BCMA molecules are required to induce cilta-cel nanobody tandem-mediated multimerization, which translates to relatively high antigen density. Further, compared to hematological malignancies such as multiple myeloma (MM), solid tumors have much more complex structures and microenvironments. Cytotoxicity, alongside immune synapse and cytotoxicity-mediating cytokine release, assays model direct cytotoxic T cell activities towards hematological malignancies agreeably, leading to successful therapies such as cilta-cel for MM. Animal models might be necessary to more reliably forecast CAR T effectiveness for solid tumors.

Cilta-cel is the first nanobody-based CAR T product approved by the US FDA. Nanobodies apparently offer greater flexibility than more conventional scFvs in CAR design primarily due to their smaller size. A tandem of nanobodies against the same antigen could engineer desirable interaction characteristics, such as those found in our case of cilta-cel for BCMA, or against different antigens for tumor targeting. The extracellular domain of BCMA is minute (54 residues). As such it is challenging to generate a cocktail of high-quality antibodies that simultaneously interact with the same BCMA_ECD_. Yet it is plausible that for larger antigens such as CD19, two nanobodies with distinct epitopes could be engineered to embrace the antigen. In relation to MM, G protein coupled receptor, family C, group 5, member D (GPRC5D) emerged more recently as another compelling target, whose expression on MM cells is independent of BCMA.^39,40^ Engineering bispecific CAR T cells with a tandem of nanobodies against BCMA and GPRC5D respectively could possibly target a more complete spectrum of MM cells, circumvent single antigen escape, and ultimately elicit deeper and more durable clinical response. Besides, the cilta-cel nanobodies exemplify a more cryptic feature that differs them from scFvs, which tend to dimerize in the absence of antigens and induce CAR clustering,^8,9^ yet this clustering might be a composite of extracellular scFv aggregation, disulfide linkage in the hinge, and transmembrane and/or intracellular association (Fig. 7). Interestingly, scFv-mediated CAR multimerization could be tweaked by the length of the linker between the variable fragments.^9^ It is conceivable that 4-1BB, as opposed to CD28, costimulation in cilta-cel might help reduce CAR T exhaustion induced by tonic signaling, as observed for scFv-based CARs that exhibit antigen-independent multimerization and clustering.^8,9^ The individual nanobodies and the tandem used in cilta-cel all behaved as monomers in our study—as observed for more soluble variants such as a V_H_H (used in cilta-cel) whose extended CDR3 shields the hydrophobic patch that normally binds a light chain—and hetero-multimerizations were dependent on the presence of the antigen BCMA.

Taken together, our results suggest antigen-induced multimerization (AIM) as a potential approach for designing future CARs: stoichiometry-determining experiments in the absence and in the presence of the antigen could be integrated in the current pipelines to help select antibodies that are otherwise similar. AIM should be contemplated in conjunction with characteristics of the antibody, such as affinity and kinetics, and other domains of the modular CAR architecture in relation to their target antigen and disease, in order to deliver optimized clinical benefits. We anticipate that, for solid tumors, patient stratification by cancer-associated antigen levels and, accordingly, differential CAR T regimens are necessary due to the tighter antigen windows in which therapies need to operate.

## Materials and methods

### Cell lines

Multiple myeloma cell lines, MM.1S and RPMI 8226, were obtained from the Cell Bank of Chinese Academy of Sciences. MM.1S-luc cells were MM.1S cells lentivirally transduced with firefly luciferase and GFP, and sorted for GFP positivity for cytotoxicity assays. The same protocol was used to generate RPMI 8226-luc and CHO-luc cells. All MM cell lines were cultured in RPMI 1640 media and 10% fetal bovine serum (FBS). LentiX-293T cells for lentiviral production were grown in Dulbecco’s Modified Eagle Medium (DMEM) medium containing 10% FBS. CHO cells were using serum-free chemically defined (CD) media (Thermo Fisher 10743029). All cell lines were cultured at 37 °C, 5% CO_2_.

### Human samples

The healthy donor peripheral blood mononuclear cells (PBMCs) were obtained from HemaCare Corporation or AllCells.

### Identification and engineering of the nanobody tandem

We immunized two llamas with purified BCMA, yielding a high-volume nanobody phage display library from which we isolated and sequenced hundreds of anti-BCMA nanobody candidates. We ranked 41 clones based on their affinities to the extracellular domain of BCMA and constructed monovalent CAR vectors with individual V_H_Hs. Despite the simplicity of the 54-residue BCMA ectodomain, we identified 8 V_H_H clones with differential epitope binding capabilities, leading to the construction of 38 multi-epitope CAR T constructs. After further cytotoxicity assessments, the Nb1-Nb2 tandem was chosen for cilta-cel due to their highest overall affinity and differential individual binding epitopes.

### Protein expression and purification

Purified Nb1, Nb2, and NbT were provided by GenScript (Nanjing, China). Briefly, the corresponding coding sequence of Nb1, Nb2, or NbT was each cloned into the pET-22b vector in frame with a carboxyl-terminal 6× His tag. These constructs were transformed into BL21 *E. coli* cells. The cells were cultivated in Luria-Bertani (LB) broth supplemented with ampicillin (100 μg/ml final concentration). The expression of nanobodies was induced with 500 µM (final concentration) isopropyl β-_D_-1-thiogalactopyranoside (IPTG) at an optical density of around 1.2, and the cultures were grown at 15 °C overnight. Cells were harvested and resuspended in a lysis buffer containing 20 mM Tris-HCl (pH 8.0) and 100 mM NaCl. After osmotic shock and centrifugation, the nanobodies were purified from the supernatant by nickel affinity chromatography and polished using a Superdex 75 column (Cytiva) in 20 mM Tris-HCl (pH 8.0) and 100 mM NaCl. BCMA_ECD_ (residues 1–54 of human BCMA; UniProt accession code: Q02223-1) was synthesized by GL Biochem (Shanghai).

### Surface plasmon resonance (SPR)

Surface plasmon resonance (SPR) binding experiments were performed using a Biacore 8 K system (Cytiva) at 25 °C in 10 mM HEPES (pH 7.4), 150 mM NaCl, and 0.05% (v/v) Tween 20. Fc-tagged and His-tagged human BCMA_ECD_ immobilized via amine coupling onto CM5 sensor chips as ligands. Five concentrations of Nb1, Nb2, or NbT obtained by two-fold gradient dilution were flowed over the chip surface as the analytes. To test whether the two proteins compete for BCMA binding, Fc-tagged human BCMA_ECD_ was captured onto a Protein A sensor chip surface via the Fc tag. To analyze the direct competitive binding characteristics of Nb1 and Nb2, Nb1/Nb2 protein was loaded followed by the mixture of Nb1 and Nb2. To analyse the binding characteristics of Nbs and BCMA ligands, Nb1/Nb2/NbT were first saturated until a binding steady state was reached. Afterward, the human BCMA ligands APRIL or BAFF were injected in the presence of Nb1/Nb2/NbT. All analyses were carried out in Biacore Insight Evaluation software (Cytiva).

### Sedimentation-velocity analytical ultracentrifugation

Sedimentation velocity was performed with an XL-I analytical ultracentrifuge (Beckman Coulter) equipped with a four-cell An-60 Ti rotor for molecular weight analysis of BCMA_ECD_, NbT, and NbT–BCMA_ECD_ complex. For other samples, including Nb1, Nb2, Nb1–BCMA_ECD_ complex, and Nb2–BCMA_ECD_ complex, an eight-cell An-50 Ti rotor was used. Protein complexes were generated by mixing Nb1, Nb2, or NbT with BCMA_ECD_ respectively in a molar ratio of 1:4, followed by incubation overnight at 4 °C. All samples were prepared in 1 mg/ml 400 μl for analysis and applied at a speed of 45,000 rpm in 20 mM Tris pH 8.0, 100 mM NaCl, at 4 °C. Absorbance scans were taken at 280 nm at the intervals of 0.003 cm size in a radial direction. The different c (s) and theoretical molecular weights were calculated by SEDFIT software.^41^

### Crystallization

The purified Nb1 or Nb2 and synthesized human BCMA_ECD_ were mixed at a molar ratio of 1:4. The mixture was incubated at 4 °C overnight and further purified using a Superdex 75 column (Cytiva). Initial crystallization trials were performed by the sitting-drop vapor diffusion method using a Mosquito (Art Robbins) crystallization robot at 20 °C. The protein solution, with a concentration of 10 mg/mL, and the reservoir solution were mixed in a 1:1 (v/v) ratio. Nb1–BCMA_ECD_ complex crystals for data collection were grown from 0.2 M ammonium sulfate, 20% w/v polyethylene glycol 3350, pH 6.0. The best crystals of the Nb2-BCMA_ECD_ complexes were grown with a well buffer containing 1.8 M sodium acetate trihydrate pH 7.0 and 0.1 M Bis-Tris propane pH 7.0. Crystals reached full size within three days and were harvested using 20% (v/v) glycerol as cryo-protectant, flash-frozen, and stored in liquid nitrogen for data collection.

### Data collection and structure determination

X-ray data were collected on beamlines BL17U1 and BL19U1 at Shanghai Synchrotron Radiation Facility (SSRF) at 100 K and at a wavelength of 0.97853 Å using a Pilatus3 6 M image plate detector. Data integration and scaling were performed using the program XDS.^42^ The structure of the Nb1-BCMA or Nb2-BCMA complex was determined by molecular replacement using the previously reported structures (PDB: 5BOP and 1XU2) as search models using the program PHASER.^43^ The output models from molecular replacement were subsequently subjected to iterative cycles of manual model adjustment with Coot^44^ and refinement was finished with Phenix.^45^ Data collection and structure refinement statistics are summarized in table S1.

### Small-angle X-ray scattering (SAXS)

Nb1–BCMA_ECD_ and Nb2–BCMA_ECD_ complex were prepared as described in crystallization. The NbT and BCMA_ECD_ were mixed at a molar ratio of 1:4. After incubation at 4 °C overnight, the mixtures were further purified by Superdex 200 column (Cytiva) gel filtration in 20 mM Tris pH 8.0, 100 mM NaCl buffer. SAXS experiments were performed at beamline BL19U2 of the National Facility for Protein Science Shanghai at SSRF. The wavelength, λ, of X-ray radiation, was set as 1.033 Å. Three concentrations were measured and SAXS data were collected at 25 °C using 60 μL sample as 20 × 1 s exposures. Data analysis was performed using BioXTAS RAW^46^ and ATSAS software package.^47^

### MM1.S proliferation assay

For proliferation assay, MM.1S cells were serum starved in RPMI 1640 media overnight. Then MM.1S cells were cultured for 3 days in RPMI 1640 media containing 2% FBS with APRIL (400 ng/mL) or BAFF (400 ng/mL) in the presence or absence of Nb1 (10 μg/mL), Nb2 (10 μg/mL), and NbT (10 μg/mL). The cells were detected by CCK-8 Assay Kit (Vazyme, A311-02) according to the protocol.

### Lentivirus package and titer

LentiX-293T cells (Clontech) were used for lentivirus production. 2 × 10^7^ LentiX-293T cells were seeded into each 15 cm dish before transfection. The next day, adherent cells with 80% confluence were accepted for transfection to obtain optimal lentivirus packaging efficiency. The transfection plasmid cocktails which included pMD2.G, pMDLg/pRRE, pRSV-Rev, and each transfer plasmid plasmids (pCDH-Nb1CAR, pCDH-Nb2CAR, pCDH-NbTCAR) were mixed by gently pipetting. PEI reagents were added to the mixture at a volume ratio of 3:1. 48 h post-transfection, the supernatant was collected and concentrated by ultracentrifugation to obtain the lentivirus. 5 × 10^6^ CHO cells in 2 mL were added to 6 well plates and serially diluted lentivirus was added into each well to initiate the transduction. 3 days later, the cells of each well were collected and stained with BCMA-FITC (ACRO, BCA-HF254) for 45 min followed by a flow cytometry assay to evaluate the virus infection titer.

### CAR T cell production

Human T cells were isolated from healthy donor PBMCs using Pan T Cell Isolation Kit (Miltenyi Biotec, 130-096-535). The purified T cells were stimulated for 48 h using T Cell TransAct reagents (Miltenyi Biotec 130-111-160) following the manual instruction. Lentivirus was mixed with T cell suspension at 5 MOI (multiplicity of infection). The bulk T cells were cultured using TexMACS GMP medium supplemented with IL-2. After seven days, CAR T cells were harvested and the transduction efficiency was assessed by fluorescence-activated cell sorter (FACS) of cells stained for BCMA-FITC (ACRO, BCA-HF254). We generated different CAR T cells, including Nb1 CAR T, Nb2 CAR T, NbT CAR T, and untreated (UT) cells, using T cells from the same healthy donor on one experimental setting.

### CAR T cell in vitro cytotoxicity co-culture assay

In cytotoxicity assays, CAR T cells were co-incubated with target cell (MM.1S-luc cells, RPMI 8226-luc cells) at an effector-to-target (E: T) ratio of 1:1 or 4:1 for 24 h. The number of CAR T cell was consistent in each group and was complemented with UT cells from the same donor according to the CAR positivity. Controls were UT cells from the same donor. After 24 h incubation in 37 °C, 5% CO_2_ cell culture incubator, 50 μL supernatants were collected for further cytokine release assay. And the cells were added with 100 μl ONE-Glo Firefly luciferase assay reagent mix (Promega, E6120) and incubated at room temperature for 1 min. The remaining living target cells were counted as a relative light unit (RLU) in a microplate reader (Tecan Spark 10 M). The cytotoxicity of CAR T on target cells was calculated with the formula as ${\rm{Viability}}=\frac{{{RLU}}_{{sample}}-{{RLU}}_{{blank}}}{{{RLU}}_{{target\; cell\; only}}-{{RLU}}_{{blank}}}$. The supernatant was collected for cytokines detection using LEGENDplex™ kits Human CD8/NK Panel kit according to the manufacturer’s protocol (BioLegend). Data were collected by using the BD LSRFortessa and analyzed with LEGENDplex v8.0. For low BCMA levels cytotoxicity assay, CAR T cells or UT cells were cocultured at the effector (CAR T or UT) to target cell (CHO-BCMA-Luc) ratio of 4:1.

### Imaging flow cytometry

The analysis of immune synapse formation between CAR T cells and MM.1S used imaging flow cytometry on Amnisr® ImageStream®X MK II. Different CAR T cells were co-cultured with MM.1S for 15 min at 37 °C with a 1:1 effector-to-target ratio. After washing cells with PBS, cells were stained with antibodies against CD3-APC-A750 (Beckman A94680), CD138 APC (Beckman A87787) for 30 min at 4 °C. After fixation and permeating, cells were stained with Phalloidin Alexa Fluor 488 (Thermo Fisher A12379) and DAPI (Santa cruz, sc-3598) at 4 °C. Data analysis was performed using Amnis IDEAS software (version 6.2). The analysis strategy we used was described previously.^48,49^ Briefly, MM.1S cells were gated first based on CD138 positive fluorescence intensity, and then conjugates of CD3, CD138 double positive cells were identified. Conjugates were used to identify adherent cells according to a strategy of Area_M01 larger than 250 units and aspect ratio lower than 0.82. For counting of immune synapse, CD3 T cells were selected to create a mask of CD3 image (“T-cell mask” = Threshold (M12, Ch12, 60)), and DAPI was selected for “valley mask” (valley (M07, Ch07,3)), and then “T-cell synapse mask” was defined (“T-cell mask” AND “valley mask”). Immune synapse formation was determined by an over 30% enrichment of F-actin in the “T-cell synapse mask”.

### Generation of CHO-BCMA-luc cells

BCMA coding sequence were retrieved from NCBI database (access number NM_001192.3) and cloned to the pUC57 vector GenScript (Nanjing, China). RNA (IVT-RNA) was prepared in vitro using the mMESSAGE mMACHINE™ T7 Transcription Kit (Invitrogen AM1344), and added poly(A) tail to RNA transcripts using Poly(A) Tailing Kit (Invitrogen, AM1350) to enhance translation initiation efficiency, then purified by the RNeasy Mini Kit (Qiagen, 74104) according to the manufacturer’s protocol. To generate CHO-BCMA-luc cells with escalating cell-surface BCMA levels, different amounts (0 μg, 0.3125 μg, 1.25 μg, or 5 μg) of prepared *BCMA* RNA were delivered into CHO-luc cells using electroporation: for each sample, 5 × 10^6^ CHO-luc cells were harvested and washed with phosphate-buffered saline once, resuspended in 120 μl electroporation buffer contained in an electroporation kit (Celetrix 1204), then transfected by electroporation at 700 V for 30 ms using a cell electroporator (Celetrix CTX-1500A).

### Quantitative analysis of cell surface BCMA antigen expression

Gradational BCMA expressed CHO-BCMA-luc cells, RPMI 8226-luc, MM.1S-luc, and five beads population of Quantum™ Simply Cellular® (Bio-rad, Cat No.815) were stained with a monoclonal anti-human CD269 (BCMA) antibody conjugated with PE (phycoerythrin) (BioLegend 357504). CHO-BCMA-luc, RPMI 8226-luc, and MM.1S-luc were stained with PE Mouse IgG2a, κ Isotype Ctrl (FC) Antibody (Biolegend, 400214) as negative control. Data were collected comprised of 10,000 cell events and 1,000 beads events. Analysis software is QuickCal v 3.0 software provided by Bangs Laboratories. The ABC value of BCMA (0 μg) is defined as ABC zero.

### Immunoblotting

The Nb1, Nb2 and NbT CAR T cells were lysed with loading buffer (Sangon Biotech C516031) with or without dithiothreitol (DTT) followed by boiling. The protein samples were separated on the 8% gel (Smart-Lifesciences SLE021) using electrophoresis and then electro-transferred onto PVDF membranes (Millipore). The membranes were blocked in a blocking buffer (5% no-fat milk in phosphate-buffered saline with Tween 20) and incubated with primary antibody overnight at 4 °C, and then an appropriate Horseradish peroxidase (HRP)-conjugated secondary antibody. The primary antibody used was MonoRab^TM^ Rabbit Anti-Camelid VHH Antibody, mAb (GenScript A01860; 1:1000 dilution). Protein expression was detected by enhanced chemiluminescence (EpiZyme SQ201). The stripping buffer (Beyotime Biotechnology P0025) was used to strip and re-probe western blot membranes. Then the membrane was blocked and incubated with GAPDH antibody (Beyotime Biotechnology AF1186; 1:2000 dilution) for detection.

### Statistical analysis

Experiments were carried out in triplicate or quadruplicate as indicated in the corresponding figure legends. For cytotoxicity assays of multiple myeloma cell lines (Fig. 6a and Supplementary Fig. 6a), cytokine releasement (Fig. 6b), and immune synapse F-actin intensity (Fig. 6d), unpaired two-tailed Student’s *t*-test was performed on each grouped sample without adjustments for multiple comparisons. For all other experiments, the data were assessed for normality and equality of variances using the Shapiro-Wilk test and Brown-Forsythe test, respectively. For normally distributed data with equal variance, the one-way analysis of variance (ANOVA) followed by Tukey’s multiple comparisons test was used to assess the differences among different groups. If the normality or equal variance conditions were violated, the Kruskal-Wallis test followed by Dunn’s multiple comparisons correction was used. Data are presented as mean ± SEM. All statistical analyses were performed using GraphPad Prism software v.9 or R.

### Supplementary information

Supplemental Material

## Data Availability

The PDB accession codes for Nb1-BCMA and Nb2-BCMA are PDB: 8HXQ and 8HXR, respectively. All other data are available in the main text or the supplementary materials.
