# Supplementary material for: Antigen-induced chimeric antigen receptor multimerization amplifies on-tumor cytotoxicity
Source: Signal Transduct Target Ther. 2023 Dec 8;8:445. doi: 10.1038/s41392-023-01686-z (PMC10703879; doi:10.1038/s41392-023-01686-z)
Supplement: Supplementary file 1 — Supplemental Material [file 41392_2023_1686_MOESM1_ESM.docx]

**Supplementary Materials for**

Antigen-induced chimeric antigen receptor multimerization amplifies on-tumor cytotoxicity

# Authors: Yan Sun^1^, Xiu-Na Yang^2^, Shuang-Shuang Yang^1^, Yi-Zhu Lyu^1,3^, Bing Zhang^2^, Kai-Wen Liu^1^, Na Li^4^, Jia-Chen Cui^1^, Guang-Xiang Huang^1^, Cheng-Lin Liu^1^, Jie Xu^1^, Jian-Qing Mi^1^, Zhu Chen^1^, Xiao-Hu Fan^5,7✉^, Sai-Juan Chen^1✉^, Shuo Chen^1,6✉^

Correspondence to Xiao-Hu Fan(frankfanwondercel@gmail.com), Sai-Juan Chen(sjchen@stn.sh.cn) & Shuo Chen(shuochen@sjtu.edu.cn)

**This PDF file includes:**

Supplementary Figure 1-6 with their legends

Supplementary Table 1- 2

**
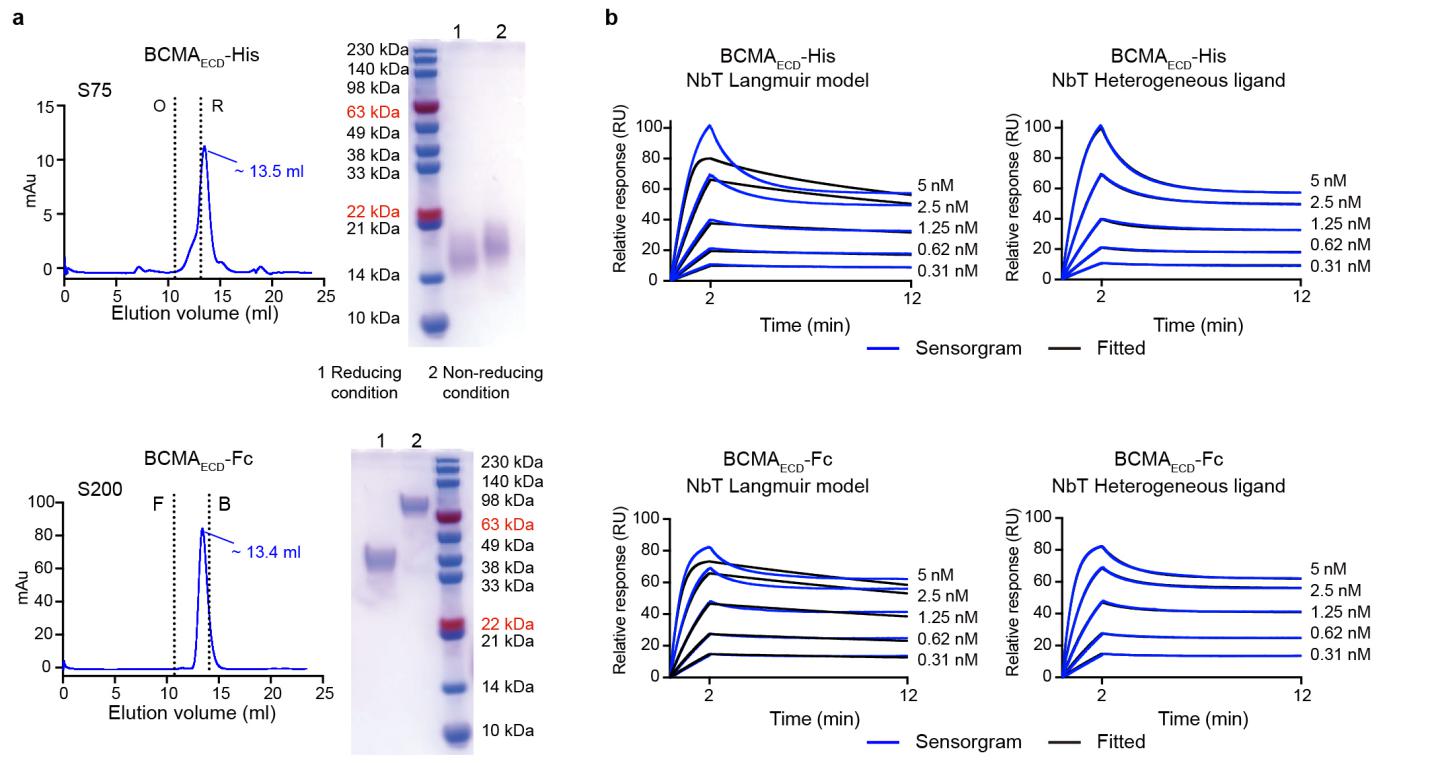
**

**Supplementary Fig 1. Surface Plasmon Resonance (SPR) analysis prompts binding model of NbT and BCMA_ECD_. a** Gel filtration and SDS-PAGE analysis of BCMA_ECD_-His and BCMA_ECD_-Fc. BCMA_ECD_-His had the same molecular weight under both reducing and non-reducing conditions, while BCMA_ECD_-Fc in non-reducing conditions presents twice that of BCMA_ECD_-Fc under reducing conditions. The elution volume of the peak is labeled. Standard molecular weight is shown as a black dotted line as a reference. F stands for Ferritin (molecular weight [Mw] 440 kDa), B indicates BSA (Mw 67 kDa), O for Ovalbumin (Mw 43 kDa), and R for Ribonuclease A (Mw 13.7 kDa). SDS-PAGE was run on the same gel and the marker lane is duplicated here. **b** Heterogeneous ligands and Langmuir kinetics model of BCMA_ECD_-His and BCMA_ECD_-Fc fit with NbT respectively. Sensorgrams are shown in blue, fitted ones in black.


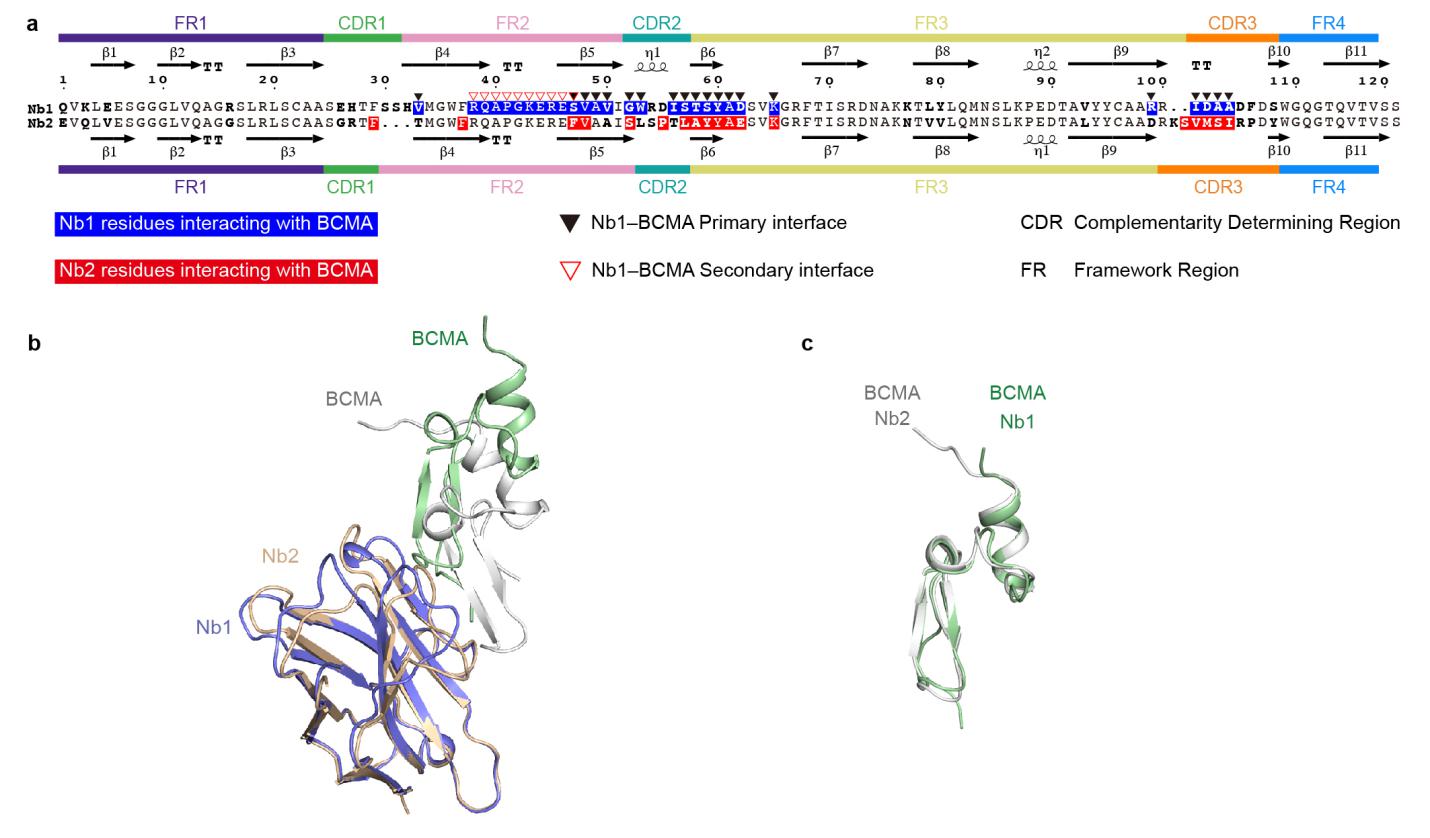


**Supplementary Fig 2. Structural basis of nanobody and BCMA complex.** **a** Sequence alignment of Nb1 and Nb2 using ESPript 3.0. Different residues between Nb1 and Nb2 are marked in bold. Nb1 residues interacting with BCMA are shown in the blue box, while Nb2 in red box. Black and white triangles indicate residues in Nb1–BCMA_ECD_ interface, respectively. The secondary structure elements of Nb1 are listed at the top of the alignment, and those of Nb2 are listed at the bottom. β-sheets, 3_10_-helix, and strict β-turns are denoted β, η, and ΤΤ, respectively. The complementarity-determining region (CDR) and framework region (FR) regions are defined according to the IgBlast tool and solved crystal structure. **b** the cartoon shows the alignment of Nb1 and Nb2. **c** the alignment of BCMA interacting with Nb1 and Nb2.


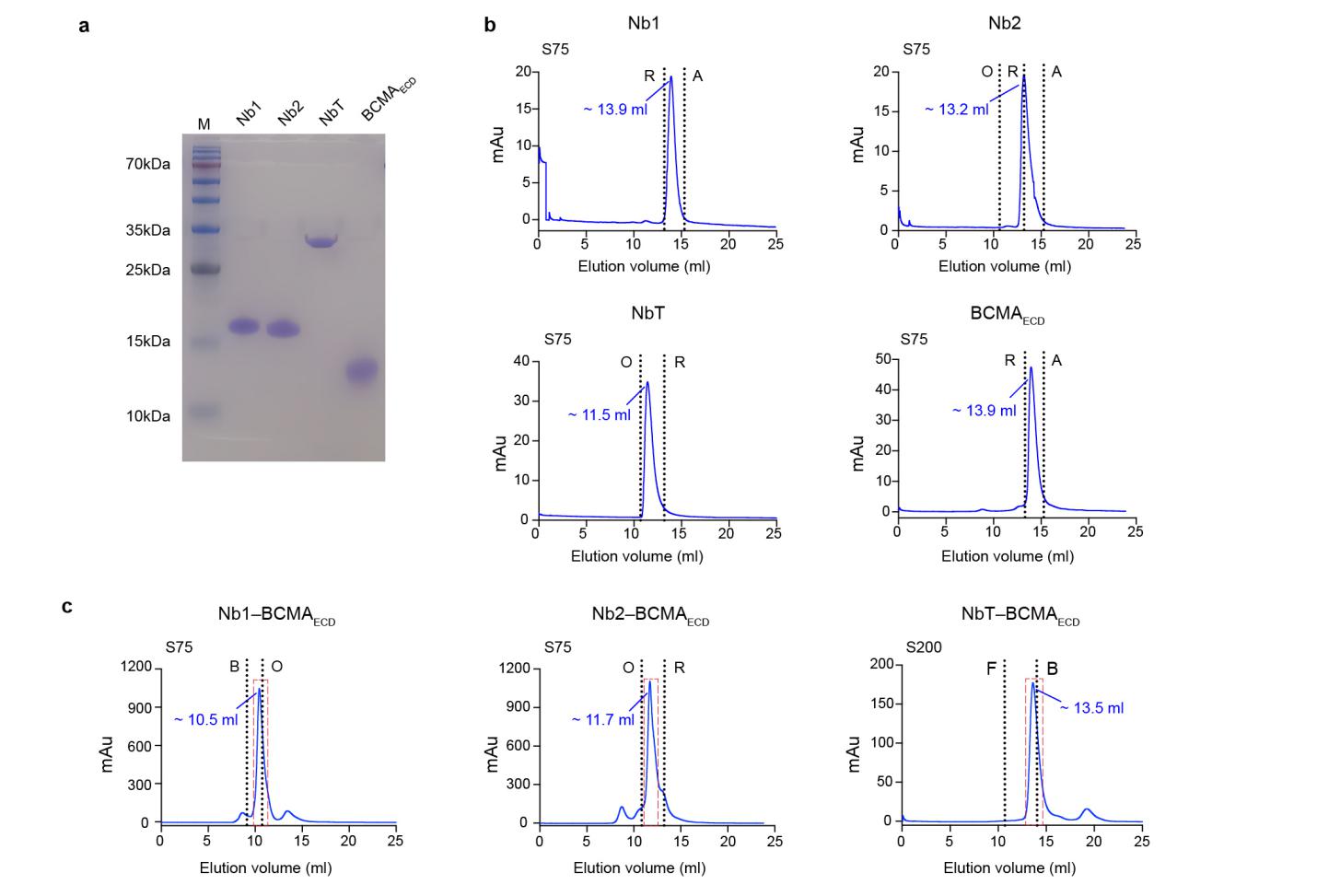


**Supplementary Fig 3. Gel filtration studies of nanobody and nanobody–BCMA_ECD_ complex. a** SDS-PAGE analysis of BCMA_ECD_, Nb1, Nb2, and NbT. **b** BCMA_ECD_, Nb1, Nb2, and NbT were purified by size-exclusion chromatography using a Superdex 75 column. The elution volume of the peak is labeled. Standard molecular weight is shown as a black dotted line as a reference. A stand for Aprotinin (Mw 6.5 kDa), others are described in supplementary Fig. S1. **c** Gel filtration of Nb1–BCMAE_CD_, Nb2–BCMA_ECD_, and NbT–BCMA_ECD_ using the indicated Superdex column. The proteins in the red dashed square were collected for crystal screening and SAXS data collection.

**
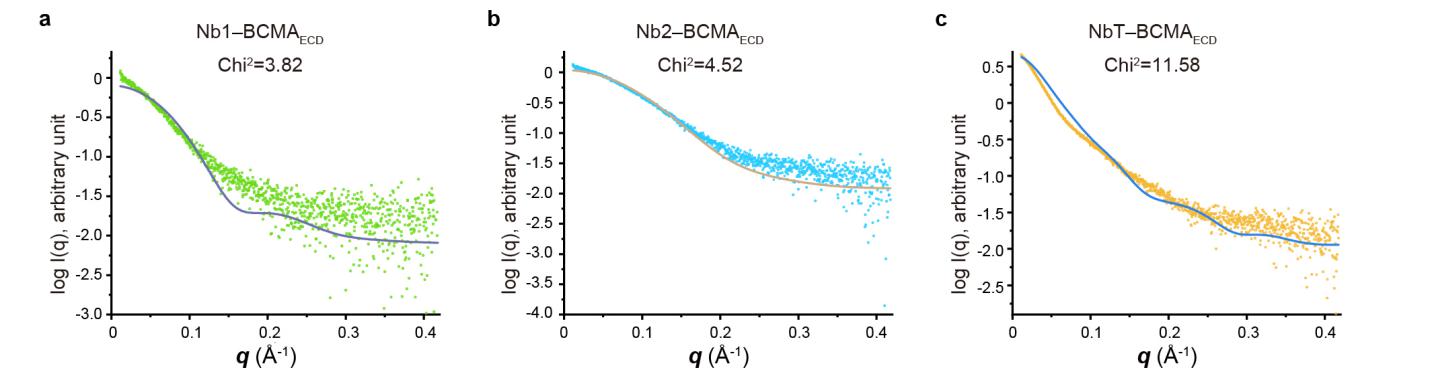
**

**Supplementary Fig 4. Comparison of the simulated atomic structures with the physiological state in solution. a** The fitting line (blue) for the Nb1–BCMA_ECD_ model is shown (green dots). The Chi^2^ equals 3.82.

**b** Model fitting (orange line) against experimental profiles (light blue dots) of Nb2–BCMA_ECD_. The Chi^2^ equals 4.52. **c** the fitting (blue line) of the NbT–BCMA_ECD_ model to the experimental profiles (orange dots) is shown. The Chi^2^ equals 11.58.


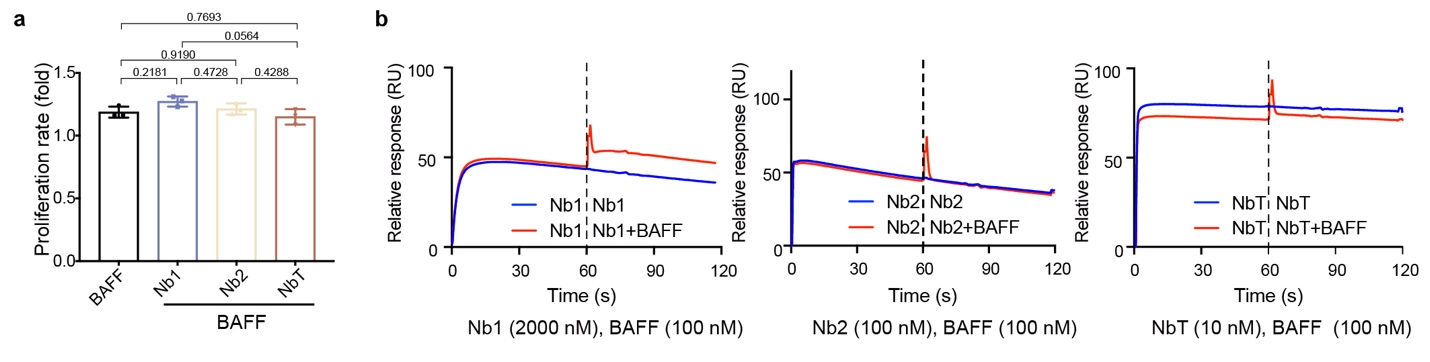


**Supplementary Fig 5. The competitive binding of nanobody and BAFF with BCMA_ECD_. a** Nbs exerted no significant effect on MM cells proliferation induced by BAFF. Data are represented as means and SEMs of n = 3 replicates per group, the one-way analysis of variance (ANOVA) followed by Tukey's multiple comparisons test was used to assess the differences among different groups. **b** Nb1 (2000nM; left), Nb2 (100 nM; middle), and NbT (10 nM; right) were probed for competitive binding against BAFF using SPR.

**
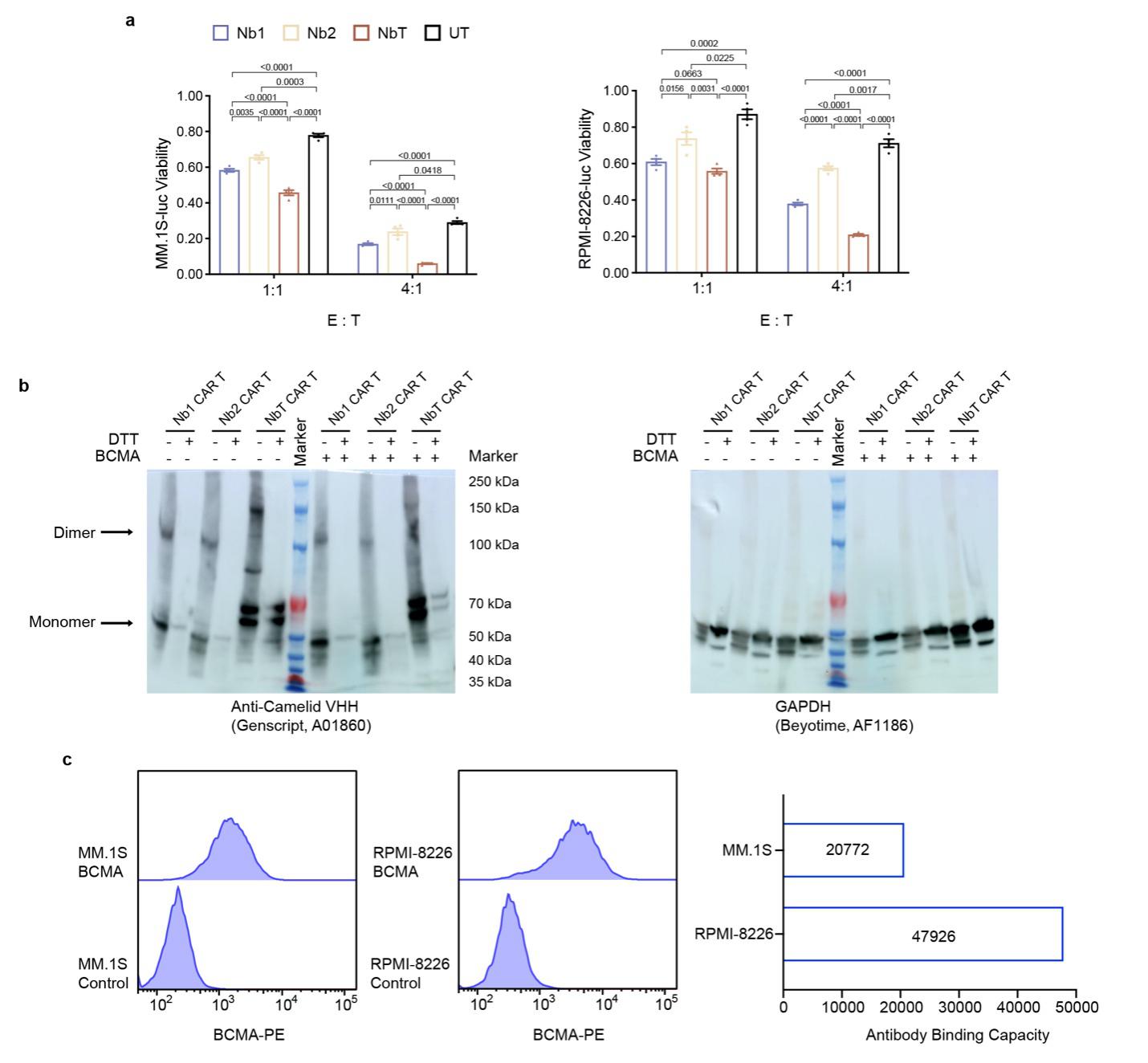
**

**Supplementary Fig 6. CAR T cell cytotoxicity against MM cell lines.** **a** Viability of MM.1S-luc and RPMI 8226-luc cells co-cultured with the indicated CAR T cells from another donor for 24 hrs at the effector: target (E: T) ratios of 1 : 1 and 4 : 1. Data are represented as means and SEMs of n = 4 replicates, using unpaired two-tailed Student’s *t* test without adjustments for multiple comparisons. **b** Representative Western blots of nanobody in CAR T cells and control (GAPDH) samples incubate with or without BCMA. Immunoblotting of disulfide-linked nanobody CARs and loading control with respectively primary and secondary antibodies (see Immunoblotting in Methods of main text for details). Synthesized BCMA was incubated with CAR T cells at 4°C for 30min. **c** Evaluation of cell-surface BCMA levels on MM.1S and RPMI-8226 cells.

**Supplementary Table 1. Crystallographic data collection and refinement statistics**

|  | Nb1–BCMA complex | Nb2–BCMA complex |
| --- | --- | --- |
| **Data collection** |  |  |
| Space group | *P4*_1_2_1_2 | *P*41 |
| Cell dimensions |  |  |
| *a*, *b*, *c* (Å) | 66.77, 66.77, 174.24 | 80.06, 80.06, 78.96 |
| α, β, γ (°) | 90, 90, 90 | 90, 90, 90 |
| Wavelength (Å) | 0.97853 | 0.97853 |
| Resolution (Å) | 50.00-2.39 (2.45-2.39) | 50.00-2.70 (2.86-2.70) |
| *R*_sym_ or *R*_merge_ | 13.6 (137.1) | 26.3 (101.9) |
| *I* / σ(*I*) | 11.33 (1.75) | 6.25 (1.65) |
| Completeness (%) | 99.6 (96.2) | 99.8 (99.6) |
| Redundancy | 12.6 (12.2) | 4.6 (4.7) |
| CC1/2 (%) | 99.4 (84.7) | 97.3 (62.3) |
|  |  |  |
| **Refinement** |  |  |
| Resolution (Å) | 47.22-2.4 | 40.03-2.7 |
| No. reflections | 16,136 | 13,778 |
| *R*_work_ / *R*_free_ | 22.6 / 27.3 | 24.0 / 28.7 |
| No. atoms |  |  |
| Protein | 2,518 | 2,446 |
| Ligand/ion |  |  |
| Water | 22 | 77 |
| *B*-factors |  |  |
| Protein | 66.25 | 30.01 |
| Ligand/ion |  |  |
| Water | 53.43 | 23.72 |
| R.m.s. deviations |  |  |
| Bond lengths (Å) | 0.006 | 0.003 |
| Bond angles (°) | 0.96 | 0.65 |
| Ramachandran plot (%) |  |  |
| Favored | 97.8 | 96.14 |
| Allowed | 1.6 | 3.22 |
| Outliers | 0.6 | 0.64 |

**Supplementary Table 2. SAXS data analysis, model fitting and software used**

| **(a) Sample details** | Nb1–BCMA_ECD_ | Nb2–BCMA_ECD_ | | NbT–BCMA_ECD_ | |  |  |
| --- | --- | --- | --- | --- | --- | --- | --- |
| Organism | Homo sapiens BCMA; Camelid nanobodies | | | | |  |  |
| Source (Catalogue No. or reference) | Synthesized BCMA_ECD;_ *E.coli* expressed nanobodies | | | | |  |  |
| UniProt sequence ID (residues in construct) | BCMA_ECD_ Q02223(1-54) | | BCMA_ECD_ Q02223(1-54) | | BCMA_ECD_ Q02223(1-54) | |  |
| Molecular mass M from chemical composition (kDa) | 40 | | 20 | | 80 | |  |
| Concentration (range/values) measured (mg/mL) | 4-5.6 | | | | |  |  |
| Solvent composition and source | 20 mM Tris, 100 mM NaCl, pH 8.0 | | | | |  |  |
| **(b) SAS data collection parameters** |  |  | |  | |  |  |
| Source, instrument and description or reference | BL19U2 BioSAXS Beamline, National Facility for Protein Science Shanghai with two detectors inline: Pilatus2M (SAXS) | | | | |  |  |
| Wavelength (Å) | 1.03 | | | | |  |  |
| Beam geometry (size, sample-to-detector distance) (H x V) mm^2^ | 330μm x 50μm (H x V), 2.74m | | | | |  |  |
| q-measurement range (Å^-1^ ) | 0.008-0.47 | | | | |  |  |
| Method for monitoring radiation damage, X-ray dose where relevant | SAXS data were collected as continuous serial exposures and scattering profiles for the passes were compared to monitor the radiation damage | | | | |  |  |
| Exposure time, number of exposures | 1 s per frame, total 20 frames | | | | |  |  |
| Sample temperature (ºC) | 25 | | | | |  |  |
| **(c) Software employed for SAS data reduction, analysis and interpretation** | | | | | | | |
| SAS data reduction | *ln*(*I*) versus *q2* using *Origin*, solvent subtraction using *PRIMUS* | | | | |  |  |
|  |  |  |  |  |  |  |  |
| Extinction coefficient estimate | http://protcalc.sourceforge.net/ | | | | |  |  |
| Basic analyses: Guinier, P(r), Vp | *PRIMUS (ATSAS 2.8.0)* ^1^ | | | | |  |  |
| *volume (e.g. Porod volume VP or volume of correlation Vc)* | *PRIMUS (ATSAS 2.8.0)* ^1^ | | | | |  |  |
| Shape/bead modelling | *DAMMIF* ^2^ | | | | |  |  |
| Molecular graphics | *PyMOL* | | | | |  |  |

| **(d) Structural parameters** |  |  |  |
| --- | --- | --- | --- |
| Guinier Analysis |  |  |  |
| Rg (Å) | 29.51±1.01 | 20.53 ± 0.57 | 47.61 ± 0.48 |
| q_min_ (Å^-1^) | 0.0108 | 0.0181 | 0.0170 |
| qRg max (q_min_=0.0071 Å^-1^) | 1.27 | 1.25 | 1.32 |
| P(r) analysis |  |  |  |
| Rg (Å) | 29.38 | 20.46 | 53.66 |
| D_max_ (Å) | 98.25 | 78.5 | 149.3 |
| q-range (Å^-1^) | 0.0108-0.2654 | 0.0181-0.3449 | 0.0170-0.1967 |
| Chi^2^ (total estimate from GNOM) | 0.921 (0.96) | 0.87 (0.91) | 0.89 (0.930) |
| Volume (e.g. VP and/or Vc) (Å^3^) | 44048 | 23177.5 | 124430 |
| **(e) Shape modelling results (a complete panel for each method)** | | | |
| DAMMIF |  |  |  |
| q-range for fitting | 0.0108-0.4176 | 0.0181-0.4176 | 0.0170-0.4176 |
| Symmetry/anisotropy assumptions | P1, none | P1, none | P1, none |
| NSD (standard deviation), No. of clusters | 0.87 (0.01),1 | 0.85 (0.02),1 | 0.91 (0.01),1 |
| χ^2 value/range | 1.098 | 0.921 | 1.606 |
| resolution (from SASRES) (Å) | 25 ± 2 | 29 ± 3 | 27 ± 2 |
| **(f) Atomistic modelling** | | | |
| CRYSOL |  |  |  |
| χ^2 value | 3.82 | 4.52 | 11.58 |
| P value | 0.05 | 0 | 0.08 |
| Predicted Rg (Å) | 29.51 | 20.53 | 42.34 |
| **(g) Data and model deposition IDs** |  |  |  |
|  | Nb1–BCMA_ECD_ | Nb2–BCMA_ECD_ | NbT–BCMA_ECD_ |
|  | SASDQV9 | SASDQW9 | SASDQX9 |

**References**

1 Franke, D. *et al.* ATSAS 2.8 : a comprehensive data analysis suite for small-angle scattering from macromolecular solutions. *Journal of applied crystallography* **50**, 1212-1225 (2017).

2 Franke, D. & Svergun, D. I. DAMMIF, a program for rapid ab-initio shape determination in small-angle scattering. *J Appl Crystallogr* **42**, 342-346, doi:10.1107/S0021889809000338 (2009).
